# Supplementary material for: The Role of Gene Expression Dysregulation in the Pathogenesis of Mucopolysaccharidosis: A Comparative Analysis of Shared and Specific Molecular Markers in Neuronopathic and Non-Neuronopathic Types of the Disease
Source: Int J Mol Sci. 2024 Dec 15;25(24):13447. doi: 10.3390/ijms252413447 (PMC11678658; doi:10.3390/ijms252413447)
Supplement: Supplementary file 1 [file ijms-25-13447-s001.zip › ijms-3353163-supplementary.pdf]

	MPS I	MPS II	MPS IIIA	MPS IIIB	MPS IIIC	MPS IIID	MPS VII
**$log_2FC > 5$**	0	0	1	0	1	0	0
**$log_2FC > 4$**	0	0	1	0	1	1	1
**$log_2FC > 3$**	2	1	2	2	3	2	6
**$log_2FC < -3$**	2	0	5	6	0	4	2
**$log_2FC < -4$**	1	0	3	1	0	2	2
**$log_2FC < -5$**	0	0	2	0	0	1	0

Figure S1. Number of transcripts with altered expression levels depending on log_2FC values in individual neuronopathic types/subtypes of MPS vs. control.

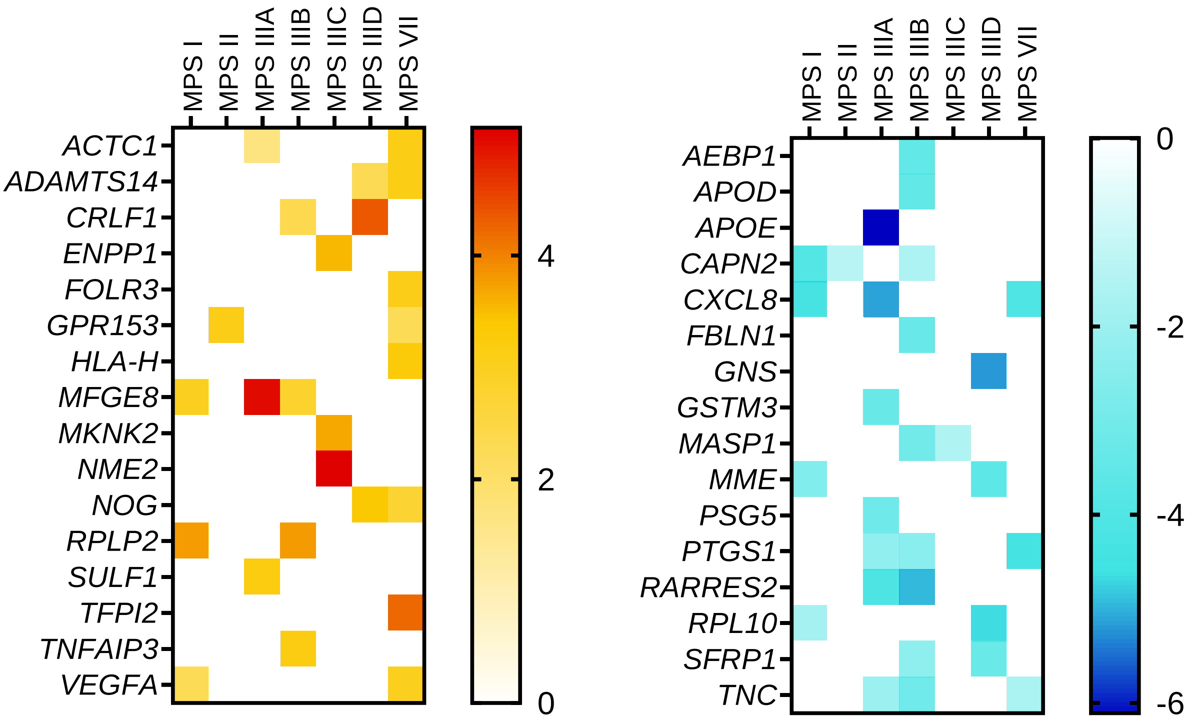

Figure S2. Genes revealing at least 3-fold changes in transcript levels between neuronopathic MPS types/subtypes compared to control cells.

Table S1. Genes revealing at least 3-fold changes (FC) in transcript levels between neuronopathic MPS types/subtypes compared to control cells, with exact FC values indicated (-, smaller changes).

log$_2$FC value of transcripts which fold change value greater than 3								
Gene name	Transcript ID	MPS I	MPS II	MPS IIIA	MPS IIIB	MPS IIIC	MPS IIID	MPS VII
ACTC1	ENST00000650163	-	-	-	-	-	-	3.15
ADAMTS14	ENST00000373207	-	-	-	-	-	-	3.15
CRLF1	ENST00000392386	-	-	-	-	-	4.38	-
ENPP1	ENST00000647893	-	-	-	-	3.57	-	-
FOLR3	ENST00000442948	-	-	-	-	-	-	3.11
GPR153	ENST00000377893	-	3.14	-	-	-	-	-
HLA-H	ENST00000383620	-	-	-	-	-	-	3,32
MFGE8	ENST00000560937	3.03	-	5.05	-	-	-	-
MKNK2	ENST00000589534	-	-	-	-	3.70	-	-
NME2	ENST00000503064	-	-	-	-	5.14	-	-
NOG	ENST00000332822	-	-	-	-	-	3.42	-
RPLP2	ENST00000530797	3.80	-	-	3.81	-	-	-
SULF1	ENST00000529041	-	-	3.22	-	-	-	-
TFPI2	ENST00000222543	-	-	-	-	-	-	4.25
TNFAIP3	ENST00000237289	-	-	-	3.20	-	-	-
VEGFA	ENST00000497139	-	-	-	-	-	-	3.03

log$_2$FC value of transcripts which fold change value less than -3								
Gene name	Transcript ID	MPS I	MPS II	MPS IIIA	MPS IIIB	MPS IIIC	MPS IIID	MPS VII
AEBP1	ENST00000450684	-	-	-	-3.43	-	-	-
APOD	ENST00000343267	-	-	-	-3.44	-	-	-
APOE	ENST00000252486	-	-	-6.11	-	-	-	-
CAPN2	ENST00000433674	-3.87	-	-	-	-	-	-
CXCL8	ENST00000307407	-4.35	-	-5.08	-	-	-	-4.04
FBLN1	ENST00000262722	-	-	-	-3.27	-	-	-
GNS	ENST00000258145	-	-	-	-	-	-5.16	-
GSTM3	ENST00000486823	-	-	-	-3.28	-	-	-
MASP1	ENST00000296280	-	-	-	-3.02	-	-	-
MME	ENST00000615825	-	-	-	-	-	-3.62	-
PSG5	ENST00000401992	-	-	-3.12	-	-	-	-
PTGS1	ENST00000223423	-	-	-	-	-	-	-4.32
RARRES2	ENST00000223271	-	-	-4.08	-4.91	-	-	-
RPL10	ENST00000406022	-	-	-	-	-	-4.61	-
SFRP1	ENST00000220772	-	-	-	-	-	-3.22	-
TNC	ENST00000350763	-	-	-	-3.08	-	-	-
